# Exhaled volatile organic compounds in patients with non-small cell lung cancer: cross sectional and nested short-term follow-up study

**DOI:** 10.1186/1465-9921-6-71

**Published:** 2005-07-14

**Authors:** Diana Poli, Paolo Carbognani, Massimo Corradi, Matteo Goldoni, Olga Acampa, Bruno Balbi, Luca Bianchi, Michele Rusca, Antonio Mutti

**Affiliations:** 1 National Institute of Occupational Safety and Prevention Research Center at the University of Parma, Via Gramsci 14, 43100 Parma, Italy; 2 Laboratory of Industrial Toxicology, Dept. of Clinical Medicine, Nephrology and Health Sciences, University of Parma, Via Gramsci 14, 43100 Parma, Italy; 3 Unit of Thoracic Surgery, University of Parma, Via Gramsci 14, 43100 Parma, Italy; 4 Respiratory Dept. and Lung Function Unit of Maugeri Foundation, Via Pinidolo 23, 25064 Gussago (Bs), Italy

## Abstract

**Background:**

Non-invasive diagnostic strategies aimed at identifying biomarkers of lung cancer are of great interest for early cancer detection. The aim of this study was to set up a new method for identifying and quantifying volatile organic compounds (VOCs) in exhaled air of patients with non-small cells lung cancer (NSCLC), by comparing the levels with those obtained from healthy smokers and non-smokers, and patients with chronic obstructive pulmonary disease. The VOC collection and analyses were repeated three weeks after the NSCLC patients underwent lung surgery.

**Methods:**

The subjects' breath was collected in a Teflon^® ^bulb that traps the last portion of single slow vital capacity. The 13 VOCs selected for this study were concentrated using a solid phase microextraction technique and subsequently analysed by means of gas cromatography/mass spectrometry.

**Results:**

The levels of the selected VOCs ranged from 10^-12 ^M for styrene to 10^-9 ^M for isoprene. None of VOCs alone discriminated the study groups, and so it was not possible to identify one single chemical compound as a specific lung cancer biomarker. However, multinomial logistic regression analysis showed that VOC profile can correctly classify about 80 % of cases. Only isoprene and decane levels significantly decreased after surgery.

**Conclusion:**

As the combination of the 13 VOCs allowed the correct classification of the cases into groups, together with conventional diagnostic approaches, VOC analysis could be used as a complementary test for the early diagnosis of lung cancer. Its possible use in the follow-up of operated patients cannot be recommended on the basis of the results of our short-term nested study.

## Background

Breath analysis seems to be a promising approach to identify new biomarkers of inflammatory and oxidative lung processes, and different volatile organic compounds (VOCs) of endogenous or exogenous origin have been analyzed to study lung diseases [1] and characterize environmental and occupational exposure to chemical pollutants [2].

During the 1970s, Pauling *et al.*[3] determined more than 200 components in human breath, some of which have subsequently been associated with different pathological conditions on the basis of their effect and/or their metabolic origin.

In 1985, Gordon *et al. *identified several alkanes and monomethylated alkanes in the exhaled air of lung cancer patients [4], an observation that aroused interest because of the possible use of exhaled biomarkers for early detection of the disease. Classical screening procedures, such as chest radiography and sputum cytology, have not decreased the number of deaths due to lung cancer [5], but promising results have recently been obtained using novel imaging techniques such as low-dose helicoidal computed tomography [6], although cost effectiveness and possible over-diagnosis seem to be serious issues. There is therefore a considerable need for non-invasive diagnostic procedures aimed at identifying lung cancer at an early stage and adding specificity to imaging techniques.

In 1999, Phillips *et al. *[7] selected 22 VOCs – mainly alkanes and benzene derivatives – to distinguish subjects with and without lung cancer, and have recently modified the VOC pattern subject to statistical analysis by reducing them to nine [8]. Selected alkanes and methylated alkanes have proved to be highly discriminating in distinguishing lung cancer patients from healthy controls, but breath analyses can be affected by both clinical and analytical confounding variables [9]. The published studies have included mixed groups of patients with primary small or non-small cell lung cancer (NSCLC) and lung metastases, and did not compare VOC levels in lung cancer patients with those in asymptomatic smokers or subjects suffering from chronic obstructive pulmonary disease (COPD), both of which may precede or be associated with the development of lung cancer and which may characterise the people undergoing screening procedures [10,11]. Furthermore, there are no data supporting the usefulness of VOC analysis in the follow-up of patients after tumour resection. Finally, only a qualitative approach has been used to identify selected VOCs, without any attempt to quantify the individual components. Actual breath concentrations could increase the statistical power of comparisons aimed at identifying differences between groups and between repeated measurements in the same individuals.

The aim of this study was to set up a new method for identifying and quantifying selected VOCs in exhaled air, and apply it to a cross-sectional study of NSCLC and COPD patients, and healthy control smokers and non-smokers, and a short-term follow-up study of patients undergoing surgery for NSCLC.

## Methods

### Study design

The design of the present study included a cross-sectional investigation during which 13 selected VOCs were measured in air exhaled by NSCLC and COPD patients, and asymptomatic control smokers and non-smokers. A subsequent nested short-term follow-up study of the NSCLC patients was carried out with repeat VOC sampling and analysis about three weeks (range 2 – 4) after they had undergone tumor resection (T_1_).

### Subjects

We enrolled 36 patients who underwent tumor resection because of histological evidence of NSCLC at the University of Parma's Department of Thoracic Surgery. The assessments of tumour size and nodes were based on the International Union Against Cancer TNM staging system [12], and all of the patients were classified as having stage Ia, Ib and IIa lung cancer. None of the patients received radiation or chemotherapy before surgery.

The study also included 25 subjects with clinically stable, mild to moderate COPD, all of whom were diagnosed on the basis of the GOLD guidelines [13]. In brief, the entry criteria, consisted of a post-bronchodilator FEV_1 _of <80% the predicted value, an FEV_1_/FVC ratio of <70%, β_2_-agonist-reversibility at baseline FEV_1 _of <200 ml and/or 15%, and the absence of clinical asthma or other significant respiratory diseases. None of them had experienced any worsening in symptoms over the previous eight weeks.

The asymptomatic controls were 35 smokers and 50 non-smokers. The smokers had to have normal spirometry values (FEV_1 _and FEV_1_/FVC) and not be suffering from chronic bronchitis; the non-smokers had to have no pulmonary symptoms or a history of pulmonary disease, and normal lung spirometry results. The smokers did not smoke for at least one hour before breath collection.

Twenty-six of the NSCLC patients agreed to repeat the breath collection during a follow-up visit 15–30 days after surgery; the other 10 were excluded from the nested follow-up study because their clinical condition had significantly worsened.

Table 1 shows the characteristics of the study subjects, all of whom gave their informed consent.

**Table 1:** Demographic characteristics of studied groups.

	NSCLC	COPD	Controls	Smokers
Subjects (n°)	36	25	50	35
Age (median, years)	67.2	70.2	55.7	54.1
Sex (male/female)	28/8	18/7	27/23	30/5
Current smokers	2	1	0	35
Ex smokers	28	21	0	0
Ever smokers	6	3	50	0
*Pack-years	20	20	n.a.	25 ± 2.6
FEV1 (% predicted)	69.8 ± 15.2	61.7 ± 13.4	105.6 ± 9.1	101.8 ± 10.2

The ex-smokers subjects had stopped smoking for at least one year. * Pack-years (mean ± SD) among current smokers. NSCLC = non-small cell lung cancer; COPD = chronic obstructive pulmonary disease; n.a. = not applicable.

### Breath collection

**Figure 1:**
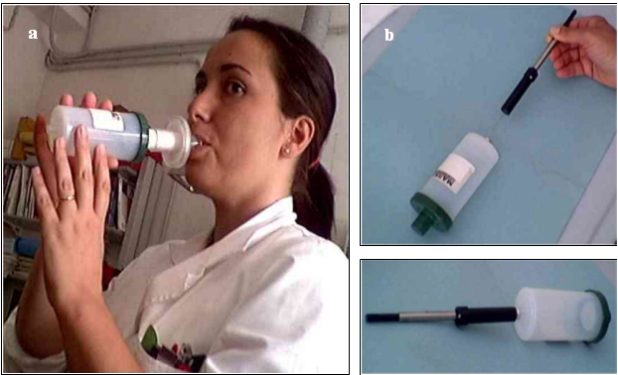
**Breath collection and VOC extraction. **The subjects performed a single slow vital capacity into a Teflon^® ^bulb (Bio-VOC^® ^breath sampler) (a) which traps the last portion of exhaled air (150 mL); the VOCs were extracted by directly inserting a 75 mm Carboxen/PDMS SPME fiber (30 min) into the bulb (b).

After carrying out a series of experiments in order to establish a reliable sampling procedure, we modified the breath sampling procedure recommended by the manufacturer of a commercially available device (Bio-VOC^® ^sampler, Markes International Ltd, Rhondda Cynon Taff, UK) (Figure 1). Briefly, after 60 minutes' rest, the subjects were asked to perform a single slow vital capacity breath into a one-way valve connected to a Teflon^®^-bulb, which traps the last portion of exhaled air (150 ml).

Twenty environmental samples were taken from the rooms in which the subjects performed the test in order to compare breath and ambient air VOC levels.

### VOC extraction and analysis

After breath collection, 1 μL of *n*-heptane-d_16 _and styrene-d_8 _methanolic solution (1.5 × 10^-5 ^M) was added to each sample as internal standard (IS) for respectively aliphatic and aromatic compounds. The exhaled VOCs and IS were extracted by means of SPME using a 75 μm Carboxen/PDMS fibre (Supelco, Bellefonte, PA, USA), which was put into the Bio-VOC^® ^breath sampler for 30 min at room temperature and then thermally desorbed in GC injection port at 280°C. The GC/MS analysis was carried out using a Hewlett-Packard HP 6890 gas chromatograph coupled with an HP 5973 mass selective detector (Palo Alto, CA, USA). The VOCs were separated on an Equity™-1 column (30 m, 0.25 mm i.d., 1.0 μm film, Supelco) and acquired in *full-scan *mode in 40–350 *m/z *range.

Thirteen VOCs (seven aliphatic and six aromatic compounds) were selected, each of which was identified by means of its mass spectrum and confirmed by comparing its retention time with that of pure standard and characteristic fragment ions; only the substances that did not interfere with co-eluting compounds were chosen.

The preliminary experiments addressed methodological issues, defined standard operating procedures, and validated analytical methods of VOC collection and analysis. The factors affect the SPME process, such as adsorption and desorption times and sampling temperature, were optimized. The extraction time profile at room temperature (22°C) was 30 min and not markedly different among the compounds. The SPME fibre was immediately transferred to the GC-injector port in order to avoid the loss of the extracted substances and avoid analyte evaporation [14]. No carry-over effects were observed when desorption was performed at 280°C for 5 min.

The method was validated by studying the linear range, and the limits of detection and precision. Linearity was established over four orders of magnitude (10^12^-10^-8 ^M, r^2^>0.98) and the limits of detection, calculated as a signal/noise ratio of about 3, was about 10^-12 ^M for all the compounds. Analytical precision, calculated as % RSD, was within 3.1–13.7% for all of the intra- and inter-day determinations on standards. The gaseous standards were directly prepared in the Bio-VOC^® ^bulb filled with helium, 1 μL of VOC methanolic standard solution, 1 μL of IS (1.5 × 10^-5 ^M), and 6 μL of deionised water. The standards were stabilised at room temperature for almost one hour and remained stable up to 60 hours.

### Statistical analysis

As the benzene and toluene levels had a log-normal distribution (the Kolmogorov-Smirnov normality test) parametric tests were used for the cross-sectional study (one-way ANOVA followed by the Games Howell post-hoc test). Non-parametric statistics (Kruskal-Wallis test followed by Dunn's Post Hoc test) were used for the other VOCs, whose distribution was not normal even after log-transformation. The cases were classified by means of multinomial logistic regression using group codes as the dependent variable and all of the VOC concentrations (except total xylenes because of their high correlation with ethylbenzene: r>0.95) as predictors. Interpretable factors based on VOC levels were obtained by means of principal component analysis (Varimax rotation with Kaiser's normalization) [15]. The Keiser Meyer Olkin (KMO) test was used to test sample adequacy (considered acceptable if the KMO constant was >0.60), and the number of factors was chosen on the basis of the flex point of the graph of decreasing eigenvalues; the percentage of variance explained was also recorded.

In the case of the follow-up study, Student's *t *test for repeated measures was applied to the benzene and toluene levels; Wilcoxon's test was used for all of the other VOCs.

A *p *value of <0.05 was considered significant for all of the statistical analyses. SPSS 13.0 (SPSS inc. Chicago, IL, USA) and PRISM 3.0 (Graphpad, San Diego, CA) were used for the statistical analyses.

## Results

Tables 2 and 3 respectively summarise the VOC levels and the statistical significances of the between-group differences. As all of the VOCs showed significant differences between at least two group pairs, the overall *p *values of the Kruskal-Wallis and ANOVA tests for individual VOCs fell between 7.5 × 10^-13 ^(for Ethylbenzene) to 1.6 × 10-^3 ^(isoprene). For these highly significant differences, adjustments for multiple testing calculated using Holm's test (less conservative than Bonferroni's test [16]) did not affect the results. The levels of 10 of the 13 substances were significantly higher in the NSCLC patients than in control non-smokers; the levels of 9 were higher in the COPD patients and control smokers than in control non-smokers.

**Table 2:** Exhaled VOC levels in studied groups

	**Controls (10^-12 ^M)**	**NSCLC (10^-12 ^M)**	**COPD (10^-12 ^M)**	**Smokers (10^-12 ^M)**
**Isoprene**	3789 (1399 – 6589)	6041 (3130 – 8863)	1758 (453 – 4981)	7243 (1361 – 16968)
**2-Methylpentane**	27.7 (3.4 – 50.3)	139.5 (65.7 – 298.8)	44.7 (21.7 – 63.8)	109.8 (62.8 – 173.5)
**Pentane**	268.0 (107.7 – 462.7)	647.5 (361.3 – 1112.5)	477.7 (261.5 – 1547.4)	511.4 (241.3 – 1128.3)
**Ethylbenzene**	13.6 (10.8 – 15.1)	24.0 (13.6 – 32.6)	51.1 (26.9 – 132.7)	39.7 (21.7 – 74.1)
**Xylenes total**	31.1 (21.1 – 56.4)	68.9 (43.6 – 108.4)	94.8 (49.7 – 131.9)	85.8 (60.1 – 185.2)
**Trimethylbenzene**	6.2 (4.7 – 11.0)	14.9 (9.3 – 22.1)	18.5 (10.4 – 25.4)	18.9 (11.9 – 44.9)
**Toluene**	80.8 (58.9 – 140.0)	158.8 (118.7 – 237.5)	158.5 (103.5 – 269.7)	453.5 (169.6 – 745.7)
**Benzene**	44.7 (27.7 – 68.6)	94.5 (62.2 – 132.2)	73.3 (51.8 – 95.4)	269.2 (84.6 – 745.1)
**Heptane**	8.4 (5.0 – 15.3)	13.5 (1.5 – 34.0)	47.3 (13.9 – 98.0)	98.0 (40.3 – 161.7)
**Decane**	208.7 (14.3 – 405.5)	568.0 (277.9 – 1321.6)	737.3 (524.6 – 1177.6)	239.2 (60.0 – 884.0)
**Styrene**	12.3 (5.3 – 21.8)	17.9 (8.5 – 37.2)	87.6 (56.0 – 148.8)	7.2 (2.8 – 41.6)
**Octane**	20.2 (4.0 – 50.8)	61.0 (22.4 – 112.9)	52.5 (31.9 – 147.2)	33.5 (19.7 – 57.8)
**Pentamethylheptane**	0.9 (0.1 – 2.6)	2.5 (1.2 – 9.7)	2.0 (1.2 – 7.6)	5.8 (1.2 – 16.5)

Concentrations expressed as median values(25^th ^-75^th ^percentile).

**Table 3:** Statistical differences between groups.

	**NSCLC vs. Controls**	**COPD vs. Controls**	**Smokers vs. Controls**	**NSCLC vs. COPD**	**NSCLC vs. Smokers**	**COPD vs. Smokers**
**Isoprene**	n.s.	n.s.	n.s.	p < 0.05	n.s.	P < 0.01
**2-Methylpentane**	p < 0.001	p < 0.05	p < 0.001	p < 0.001	n.s.	P < 0.05
**Pentane**	p < 0.001	p < 0.05	p < 0.05	n.s.	n.s.	n.s.
**Ethylbenzene**	p < 0.01	p < 0.001	p < 0.001	p < 0.05	n.s.	n.s.
**Xylenes total**	p < 0.001	p < 0.001	p < 0.001	n.s.	n.s.	n.s.
**Trimethylbenzene**	p < 0.01	p < 0.001	p < 0.001	n.s.	n.s.	n.s.
**Toluene**	p < 0.001	n.s.	p < 0.001	n.s.	p < 0.001	P < 0.01
**Benzene**	p < 0.001	n.s.	p < 0.001	n.s.	p < 0.001	P < 0.05
**Heptane**	n.s.	p < 0.01	p < 0.001	n.s.	p < 0.001	n.s.
**Decane**	p < 0.001	p < 0.01	n.s.	n.s.	n.s.	n.s.
**Styrene**	n.s.	p < 0.001	n.s.	p < 0.001	n.s.	P < 0.001
**Octane**	p < 0.001	p < 0.01	n.s.	n.s.	n.s.	n.s.
**Pentamethylheptane**	p < 0.001	n.s.	p < 0.001	n.s.	n.s.	n.s.

The significance of the multiple comparisons inside the individual univariate tests. ANOVA followed by Games Howell Post Hoc test for benzene and toluene, Kruskal-Wallis test followed by Dunn's Post Hoc test for all the other VOCs were performed.

The NSCLC patients had significantly higher 2-methylpentane and isoprene levels and significantly lower ethylbenzene and styrene levels than the COPD patients, and significantly lower benzene, heptane and toluene levels than the control smokers. In comparison with the control smokers, the COPD patients had lower 2-methylpentane, benzene and toluene levels, and higher styrene levels.

Exhaled breath of non-smoking controls had higher levels of isoprene and heptane than the environmental air, whereas NSCLC and COPD patients and control smokers showed higher levels of almost all substances (data not shown).

Principal component analysis (table 4), with a KMO constant of 0.83, distinguished three factors with eigenvalues >1, of which the third was the flex point of the graph of decreasing eigenvalues. The first grouped benzene, heptane, toluene, ethylbenzene, trimethylbenzene with an explained variance of 27.5% (total xylenes were excluded because of their high correlation with ethylbenzene: r>0.95); the second grouped octane, styrene, pentamethylheptane and decane with an explained variance of 20%, and the third grouped pentane, isoprene and methylpentane with an explained variance of 19%. The total explained variance of the model was therefore 66.5%.

**Table 4:** Principal Components analysis of variables.

		**Factors**		
		
	**Group**	**1**	**2**	**3**
**Isoprene**	1			0.797
**2-Methylpentane**	1			0.562
**Pentane**	1			0.531
**Ethylbenzene**	2	0.851		
**Trimethylbenzene**	2	0.794		
**Toluene**	2	0.773		
**Benzene**	2	0.728		
**Heptane**	2	0.629		
**Decane**	3		0.878	
**Styrene**	3		0.704	
**Octane**	3		0.643	
**Pentamethylheptane**	3		0.592	

**Figure 2:**
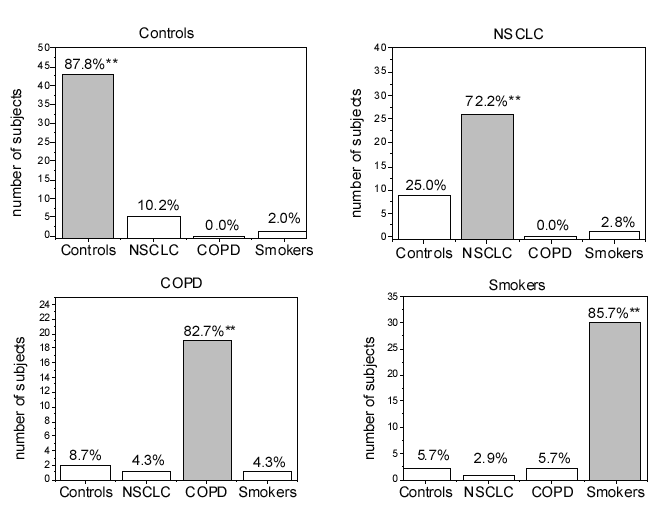
Classification of cases with multinomial logistic regression analysis. ** Correctly classified cases. 82.5% of the subjects were correctly classified.

In order to test the discriminant power of the exhaled VOC pattern, a multinomial logistic regression was made using the coding group as the output variable and the concentration of all of the VOCs except total xylenes as predictors: concentrations were used because they are direct measures with an intrinsic experimental error and therefore more appropriate than the ratio between exhaled breath and air VOC concentration, a function derived from two different experimental measures by means of mathematical manipulations. Figure 2 shows the correct classification of cases into four groups as the Cox and Snell pseudo R-square of the model was 0.83 (goodness-of-fit test). In general, 82.5% of subjects were correctly classified: a maximum of 87.8% for control non-smokers and a minimum of 72.2% for the NSCLC patients. Analysis of residuals did not reveal any particular cases with an undue influence on the model or the overall classification. On the basis of these results, the overall sensitivity (calculated as NSCLC true positive/ true positive + false negative) was 72.2% and overall specificity (calculated as NSCLC true negative/ true negative + false positive) was 93.6%.

In the follow-up study of the NSCLC patients, only isoprene and decane significantly decreased after surgery (*p *< 0.05, table 5).

**Table 5:** VOCs levels at T_0 _(before surgery) and T_1 _(after surgery).

	**T_0_**	**T_1_**
**Isoprene**	**6121 (4069–9031)**	*4125 (2415–7407)
**2-Methylpentane**	139.5 (68.8–291.6)	123.5 (81.1–227.6)
**Pentane**	647.5 (388.5–1013)	529.5 (329.6–960.0)
**Ethylbenzene**	24.0 (14.8–28.0)	19.7 (15.7–34.5)
**Xylenes total**	69.0 (45.8–105.6)	67.8 (51.2–129.4)
**Trimethylbenzene**	15.2 (10.1–22.3)	13.2 (10.2–22.5)
**Toluene**	161.9 (118.7–232.5)	160.3 (119.0–232.7)
**Benzene**	95.7 (62.9–132.2)	99.6 (60.0–119.2)
**Heptane**	15.1 (0.9–34.6)	18.7 (9.5–39.5)
**Decane**	**625.0 (322.6–1392)**	*443.0 (197.0–920.7)
**Styrene**	22.1 (11.5–38.1)	18.0 (12.1–43.1)
**Octane**	65.7 (45.8–131.4)	49.7 (28.5–102.5)
**Pentamethylheptane**	2.6 (1.7–10.0)	2.5 (1.1–8.8)

* means a statistically significant difference (*p *< 0.05). The data are expressed as median (25^th ^-75^th ^percentile).

## Discussion

Non-invasive diagnostic strategies aimed at identifying biomarkers of early lung cancer probably require the use of a panel rather than single substances [17]. The main finding of our study was that none of selected VOCs alone distinguished the NSCLC patients from the other study groups (i.e. non of them was a specific biomarker of NSCLC), but overall VOC concentrations were highly discriminant (>70%). Owing to the limited sensitivity and specificity of VOC analysis, a NSCLC diagnosis only based only VOC concentrations in exhaled breath cannot be recommended at this stage. We did not calculate positive and negative predictive values, as they are highly dependent on the prevalence of the condition being examined in the population at hands. Owing to the low prevalence of NSCLC even in selected groups at high risk, the positive predicted value of exhaled VOCs is expected to be low, and should probably be used to rule out, rather than to confirm NSCLC in subjects with suspect nodules.

Moreover, exhaled breath analysis is a particularly interesting strategy but is still hampered by the lack of a standardised breath collection system and putative exhaled biomarkers.

Our simple method of breath collection has a number of advantages: *i) *it samples a fixed volume of air and discards anatomic dead space air; *ii) *its fixed resistance allows a reasonably constant expiratory flow; *iii) *it has no carry-over effects and permits the addition of internal standards to the breath samples, which improves data reproducibility; and *iv) *it is a well-tolerated, suitable for screening purpose, and also applicable to difficult clinical and psychological conditions such as those observed in NSCLC patients.

Further studies are needed to evaluate the VOC levels obtained from repeated expirations or tidal breathing, but the collection procedures require respiratory devices equipped with instruments that control ventilatory pattern [18], and this may limit their widespread application.

We selected 13 VOCs from the chromatographic profile of exhaled breath on the basis of the detectability of the peak and their biological significance, ten of which have been previously used for discriminant lung cancer analysis by Phillips *et al. *[7]; the other three were markers of oxidative stress such as pentane with its methylated form (2-methylpentane), and toluene, which is closely related to cigarette smoke.

The fact that we identified fewer VOCs than Phillips *et al. *[7] may have been partially due to differences in our breath sampling procedures: rather than concentrating the breath sample in a sorbent trap [19], we collected breath VOCs from a single expiration and extracted them using SPME fibre. The SPME technique may be less sensitive, but has the advantages of not requiring sample preparation or any specific equipment for GC analysis [20]; furthermore, it allowed us to measure most of the substances of interest proposed in the literature. Another reason for the difference in VOC identification may be the different clinical characteristics of lung cancer patients: we enrolled early-stage NSCLC patients because they may benefit more from early detection strategies.

There were no significant differences between the level of most of the VOCs in the exhaled air of the control non-smokers and those in the ambient air, which suggests that ambient levels may influence the VOCs exhaled by healthy non-smokers (data not shown). However, the VOC levels in diseased patients were not explainable solely by ambient VOC concentrations during breath collection, because the samples of all of the study subjects were collected in the same place. The NSCLC and COPD patients and the control smokers had generally higher levels of all of the exhaled VOCs than the control non-smokers (except for isoprene in the COPD group), which reflects differences in exhaled air composition due to pathological conditions or smoking rather than environmental contamination.

Various approaches have been adopted in an attempt to distinguish endogenous substances from exogenous contaminants, such as correcting exhaled VOC concentrations by subtracting inspiratory VOC levels or by calculating alveolar gradients [7]. However, although these methods are easy to perform, they do not take into account the complexity of pulmonary adsorption and exhalation of volatile substances [2].

Although the exact origin of exhaled VOCs remains to be demonstrated, principal components analysis (PCA) factorised the compounds into three groups (table 4) and suggests some fascinating hypoteses. It may be particularly relevant in distinguishing substances of endogenous origin from those influenced by confounding factors mainly related to tobacco smoke.

Isoprene, pentane and 2-methylpentane are grouped together (group 1, factor 3). These substances can be considered mainly endogenous compounds even though pentane and its methylated forms are also present in vehicle engine exhausts [21] and isoprene is also a constituent of tobacco smoke [22]. In humans, isoprene is formed from acetilCoA and is the basic molecule in cholesterol biosynthesis [23], and pentane comes from human lipid peroxidation [24]. The grouping of these with 2-methylpentane is in line with the results of a previous study that considered methylated alkanes as a secondary product of human oxidative stress [25], although the exact source of methylated alkanes is still debated [26].

Of the group 1 substances, 2-methylpentane levels were higher in NSCLC patients than in the control non-smokers and COPD patients, which suggests its potential usefulness in screening procedures (probably in combination with other relevant biomarkers). In line with previous observations [27], pentane levels were higher in the exhaled air of the patients with NCSLC and COPD and asymptomatic smokers than in the control non-smokers, but did not differentiate the first three groups from each other.

Also in line with previously published studies [27,28], isoprene levels were significantly higher in the breath than in the environmental samples (data not shown), and higher in the NSCLC patients and control smokers than in the COPD patients. The between-group differences are difficult to interpret, but are probably related to the moderate effect of cigarette smoke on isoprene levels, and partially to the lung destruction (emphysema) often affecting COPD patients. In this regard, although no studies have compared breath isoprene levels in NSCLC and COPD patients, lower levels have been observed in the exhaled breath of patients with acute respiratory distress syndrome (ARDS) in comparison with those without ARDS [29].

The substances belonging to group 2 (factor 1) could be classified mainly as smoking-related exogenous compounds because their levels were higher in the control smokers than control non-smokers. Ethylbenzene may be of particular interest because of its ability to distinguish NSCLC and COPD patients, and control non-smokers.

The substances belonging to group 3 (factor 2) are heterogeneous and it is therefore more difficult to interpret the between-group differences in the levels of the individual substances.

The results of the VOC analysis of our nested short-term follow-up study of surgically treated NSCLC patients showed that only isoprene and decane levels significantly decreased after surgery (Table 5), thus indicating that breath VOC analysis cannot be recommended as a short-term follow-up procedure in such patients.

## Conclusion

Although none of the individual exhaled VOC alone was specific for lung cancer, a combination of 13 VOCs does allow the classification of cases into groups. Exhaled VOC analysis may therefore be useful in improving the specificity and sensitivity of conventional diagnostic approaches to lung cancer. However, these findings will require validation in larger clinical studies.

## List of abbreviation used

COPD = Chronic Obstructive Pulmonary Disease; GC/MS = Gas Chromatography/Mass Spectrometry; IS = internal standard; NSCLC = Non-Small Cells Lung Cancer; PCA = Principal Components Analysis; SPME = Solid Phase Microextraction; VOC = Volatile Organic Compound; trimethylbenzene = 1,2,4- trimethylbenzene; pentamethylheptane = 2,2,4,6,6-pentamethylheptane.

## Competing interests

All authors excluded any competing interest.

## Authors' contributions

DP: substantial contribution to conception and design, acquisition of data, analysis and interpretation of data, involved in drafting the articles.

PC: substantial contribution to conception and design, collection of samples, revision of the draft critically for important intellectual content.

MC: substantial contribution to conception and design, analysis and interpretation of data, involved in drafting the articles.

MG: substantial contribution to conception and design, statistical analysis and interpretation of data, involved in drafting the articles.

OA: collection of samples, revision of the draft critically for important intellectual content.

BB: substantial contribution to conception and design, collection of samples, revision of the draft critically for important intellectual content.

MR: substantial contribution to conception and design, collection of samples, revision of the draft critically for important intellectual content.

AM: substantial contribution to conception and design, statistical analysis and interpretation of data, involved in drafting the articles, final approval of the version to be published.
